# Molecular detection of pathogens from ticks collected from dogs and cats at veterinary clinics in Finland

**DOI:** 10.1186/s13071-023-05864-4

**Published:** 2023-09-13

**Authors:** Fathiah Zakham, Essi M. Korhonen, Petteri T. Puonti, Robert S. Castrén, Ruut Uusitalo, Teemu Smura, Ravi Kant, Olli Vapalahti, Tarja Sironen, Paula M. Kinnunen

**Affiliations:** 1 Department of Virology, Faculty of Medicine, University of Helsinki, Helsinki, Finland; 2 Department of Veterinary Biosciences, Faculty of Veterinary Medicine, University of Helsinki, Helsinki, Finland; 3 Faculty of Pharmacy, University of Helsinki, Helsinki, Finland; 4 Department of Geosciences and Geography, University of Helsinki, Helsinki, Finland; 5 Clinical Microbiology, HUS Diagnostic Center, University of Helsinki and Helsinki University Hospital, Helsinki, Finland; 6 Companion Animal Business Unit, Nordic Cluster, MSD Animal Health, Espoo, Finland

**Keywords:** *Anaplasma* spp., *Babesia* spp., *Borrelia* spp., *Candidatus* Neoehrlichia mikurensis, *Ehrlichia canis*, Ixodid ticks, Next-generation sequencing, One Health, PCR, Tick-borne encephalitis virus

## Abstract

**Background:**

Ticks carry microbes, some of which are pathogenic for humans and animals. To assess this One Health challenge, 342 ticks were collected from pet dogs and cats at 10 veterinary clinics in Finland as part of the European project “Protect Our Future Too”.

**Methods:**

The tick species were identified, and ticks were screened with quantitative PCR (qPCR) for tick-borne pathogens, including *Borrelia burgdorferi* sensu lato, *Borrelia miyamotoi*, *Ehrlichia canis*, *Anaplasma* spp., *Candidatus* Neoehrlichia mikurensis, tick-borne encephalitis virus (TBEV), and *Babesia* spp. For comparison, a subset of tick DNA (20 qPCR-positive samples) was analysed with 16S next-generation sequencing (NGS).

**Results:**

Most ticks were *Ixodes ricinus* (289, 84.5%), followed by *Ixodes persulcatus* (51, 14.9%). One hybrid tick (*I. ricinus*/*I. persulcatus*, 0.3%) and one *Rhipicephalus sanguineus* tick (0.3%) were identified. We found one or more of the analysed pathogens in 17% (59/342) of the ticks. The most prevalent pathogen was *B. burgdorferi* s.l. (36, 10.5%), followed by *Anaplasma phagocytophilum* (12, 3.5%), *B. miyamotoi* (5, 1.5%), *Babesia venatorum* (4, 1.2%), and TBEV (1, 0.3%). *Candidatus* Neoehrlichia mikurensis DNA was amplified from three (0.9%) ticks. *Ehrlichia canis* was not detected. In the 16S NGS, six samples produced enough reads for the analysis. In these six samples, we confirmed all the positive qPCR findings of *Borrelia* spp. and *Ca.* N. mikurensis.

**Conclusions:**

The high prevalence of pathogenic microorganisms in the ticks of this study emphasizes the importance of awareness of ticks and tick-borne diseases and prevention. Furthermore, the results show that veterinary surveillance can facilitate early detection of tick-borne pathogens and new tick species and draw attention to possible co-infections that should be considered both in symptomatic humans and animals after tick bites.

**Graphical Abstract:**

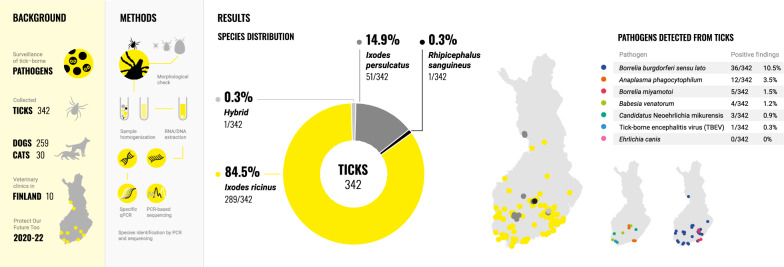

**Supplementary Information:**

The online version contains supplementary material available at 10.1186/s13071-023-05864-4.

## Background

Dogs and cats are the most common pets, and exposure to ticks is inevitable when these animals engage in outdoor activities. Ticks ectoparasitize their hosts, including pets, at each stage of their life cycle and may transmit severe bacterial, parasitic, and viral pathogens. These pathogens can induce behavioural changes in ticks and affect their phenotypic characteristics, making them more active in host-seeking and more resistant against extreme environmental events (e.g., desiccation and cold), which leads to increased fitness and survival [1]. The transmission of pathogens from ticks to hosts depends mainly on the duration of host attachment. For instance, tick-borne encephalitis virus (TBEV) can be transmitted within 60 min of attachment. However, other microorganisms require a longer period [2]. Transmission times may vary considerably between different tick vectors, pathogens, pathogen quantity, host species, and conditions; successful pathogen transmission may require feeding longer than 48 h [3, 4].

Tick-borne infections represent a One Health concern. Without proper protection, tick bites may lead to transmission of tick-borne microorganisms to the animals or the pet owners, which may cause serious and even life-threatening illness. In the northern part of Europe, the most common tick-borne pathogens of zoonotic potential are *Borrelia* spp., *Anaplasma phagocytophilum*, and TBEV. However, *Rickettsia* spp., *Candidatus* Neoehrlichia mikurensis, and *Babesia* spp. are also reported [5, 6]. Further, polymicrobial infections can occur, as ticks can carry multiple pathogens simultaneously. This can complicate the diagnosis and management of some cases, especially due to the lack of broad-spectrum diagnostic tools for routine testing and the limited therapeutic options [5, 6].

In Finland, tick-borne infections are mainly transmitted by *Ixodes ricinus* (dominant in southern Finland) and *Ixodes persulcatus* (dominant mostly in northern Finland) ticks [7, 8]. Distribution of these ticks is determined by climatic and environmental conditions and animal hosts. In addition, migratory birds and relocated dogs occasionally carry new and rare tick species, which may become endemic in the future climate [9, 10]. According to the Finnish Institute for Health and Welfare, most of the diagnosed and registered human tick-borne infections are Lyme borreliosis (LB; approximately 2000–2500 cases annually) and tick-borne encephalitis (TBE; 151 cases in 2021) [11]. In contrast, a seroprevalence study of Finnish pet dogs suggested that *A. phagocytophilum* (5.3%) is the most common tick-borne pathogen that dogs encounter, followed by *Borrelia burgdorferi* (2.9%) and *Ehrlichia canis* (0.3%) [12]. Another study showed a TBEV seroprevalence of 40% in the Åland Islands and 6% on the south-western archipelago of Finland in samples collected from dogs, which indicates that exposure varies according to geographical location of the animals [13]. This is consistent with the tick abundance and infection prevalence of ticks in a given area.

Several European countries have reported an increase of tick-borne infections, occurring mainly due to changes in climatic and environmental conditions, host reservoir densities, and exposure of human populations [14]. In this context, there is a crucial need for understanding the circulation and the diversity of tick-borne pathogens causing diseases in animals and humans. Pets, particularly dogs, can be used as sentinels for tick-borne diseases. Tick surveillance and analysis in pets can help in estimating the potential risk of these often zoonotic pathogens [15].

As part of the European project “Protect Our Future Too”, which is focused on studying the effects of climate change on pets [16], we performed a study on tick-borne pathogens of veterinary and medical importance in Finland.

## Methods

### Tick collection

**Fig. 1:**
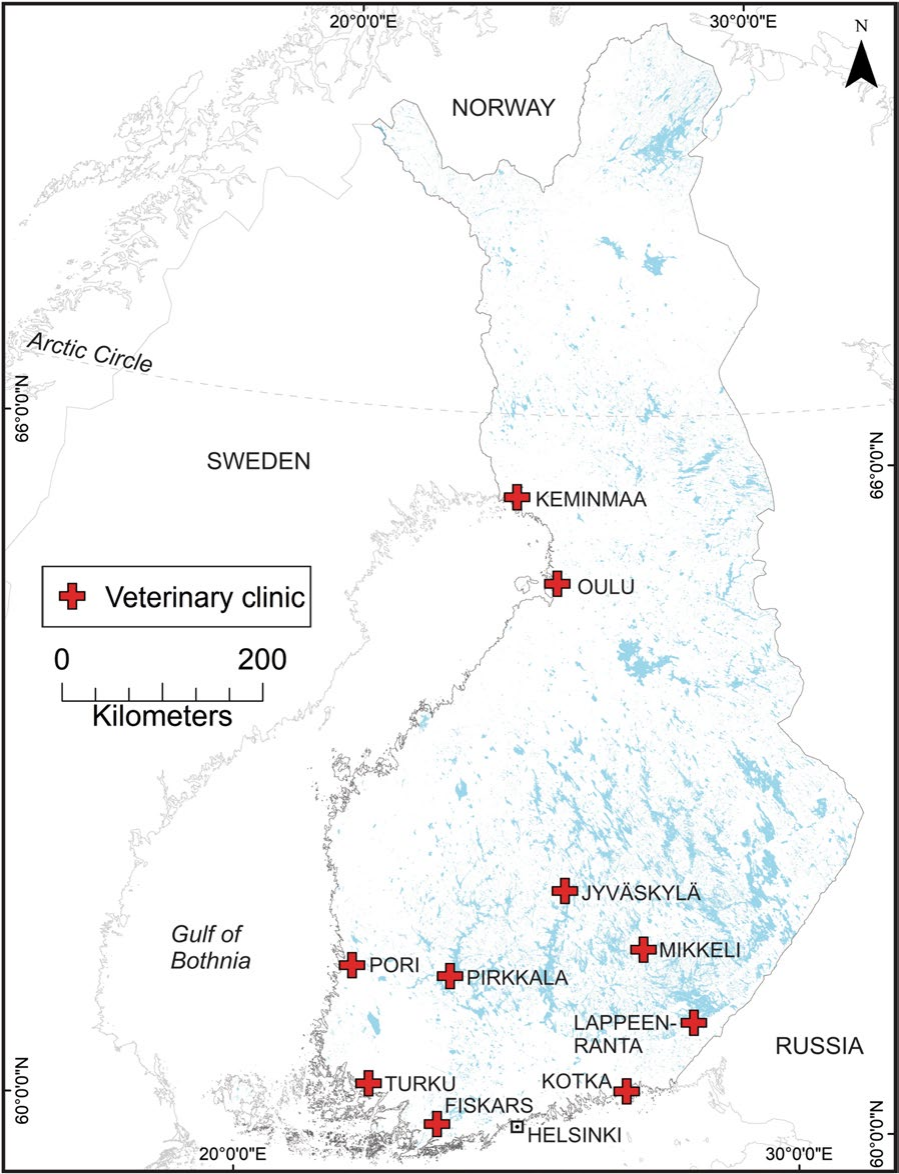
The locations of veterinary clinics. Map indicates the location of the veterinary clinics, which collected ticks from pets

Ticks were collected from pet dogs and cats at 10 veterinary clinics across Finland (Fiskars, Kotka, Turku, Lappeenranta, Pori, Pirkkala, Mikkeli, Jyväskylä, Oulu, and Keminmaa; Fig. 1). Tick collections were performed over a period of 12–16 months by each clinic between June 2020 and November 2021 (Fig. 1). Ticks were removed with Safeguard cards (one for each pet), transferred by pinching the tick legs with tweezers into a sterile tube, and stored refrigerated in RNAlater (Thermo Scientific) until they were shipped to the University of Helsinki for species identification and pathogen screening. Data were recorded upon arrival of a subset of ticks and included information on animal species from which ticks were removed, locality, traveling history of the pet, whether ticks were attached, level of engorgement, developmental status, and sex of ticks.

### DNA and RNA extraction

Ticks were homogenized on 96-well plates or in 1.5-ml tubes depending on the number of samples prepared. For homogenization, the ticks were placed individually in the tubes or wells with a 3-mm metal bead and sterile sand. A volume of 400 µl of Dulbecco's phosphate-buffered saline solution with 0.2% bovine serum albumin was added into each tube. Ticks were then homogenized with Qiagen TissueLyser II at a frequency of 30 Hz for 3 min. RNA extraction was performed using a QIAamp Viral RNA Kit (Qiagen) with either the spin protocol (small sample sizes) or the QIAcube (larger sample sizes). DNA extraction was performed on the remaining homogenates with a GeneJET Genomic DNA Purification Kit (Thermo Scientific).

### Tick species identification

Identification of tick species was performed primarily by species-specific probes in duplex polymerase chain reaction (PCR) targeting the internal transcribed spacer 2 (*ITS2*) gene as previously described (Table 1) [17] and by sequencing the same gene [18]. Ticks that were morphologically identified as something other than *Ixodes* spp. were investigated with PCR and sequencing targeting the *ITS2* [18] and cytochrome *c* oxidase subunit 1 (*COX1*) genes [19, 20]. The species were verified based on the sequences using the BLAST program available at the National Center of Bioinformatics (NCBI).

**Table 1:** Detailed description of primers and probes used in this study

Targeted parasite/pathogen	Gene	Primer/probe	Sequence	Refs.
*Ixodes* spp.	*ITS2*	IXO-I2-F4	TCTCGTGGCGTTGATTTGC	[17]
*Ixodes* spp.		IXO-I2-R4	CTGACGGAAGGCTACGACG	
*I. persulcatus*		Ipe-I2-P4	[FAM]-TGCGTGGAAAGAAAACGAG-[BHQ1]	
*I. ricinus*		Iri-I2-P4	[Hex]-TGCTCGAAGGAGAGAACGA-[BHQ1]	
PCR-based sequencing for tick species barcoding	*ITS2*	dITS29	CCTTCCCGTGGCTTCGTCTGT	[18]
		rITS800	GGGGGTTGTCTCGCCTGATGT	
*R. sanguineus* sensu lato	*COX1*	S0725	TACTCTACTAATCATAAAGACATTGG	[19, 20]
		S0726	CCTCCTCCTGAAGGGTCAAAAAATGA	
*B. burgdorferi* s.l.	*ospA*	Bbsl-ospA-F	AATATTTATTGGGAATAGGTCTAA	[53]
		Bbsl-ospA-R	CACCAGGCAAATCTACTGA	
		Bbsl-ospA-P	[FAM]-TTAATAGCATGTAAGCAAAATGTTAGCA-[DDQ1]	
*B. miyamotoi*	*flaB*	Bm-fla-F	AGAAGGTGCTCAAGCAG	[54]
		Bm-fla-R	TCGATCTTTGAAAGTGACATAT	
		Bm-fla-P	[FAM]-AGCACAACAGGAGGGAGTTCAAGC-[BHQ1]	
*Anaplasma* spp.	*Msp2*	ApMSP2-F	ATGGAAGGTAGTGTTGGTTATGGTATT	[55]
		ApMSP2-R	TTGGTCTTGAAGCGCTCGTA	
		ApMSP-P	[CY5]-TGGTGCCAGGGTTGAGCTTGAGATTG-[BBQ650]	
*Babesia* spp.	18S rRNA	Bab18S-F	CAGCTTGACGGTAGGGTATTGG	[56]
		Bab18S-R	TCGAACCCTAATTCCCCGTTA	
		Bab18S-P	[HEX]-CGAGGCAGCAACGG-[BHQ1]	
*Ca.* Neoehrlichia mikurensis	*GroEL*	CNeGroEL-F	CCTTGAAAATATAGCAAGATCAGGTAG	[57]
		CNeGroEL-R	CCACCACGTAACTTATTTAGCACTAAAG	
		CNeGroEL-P	[FAM]-CCTCTACTAATTATTGCWGAAGATGTAGAAGGTGAAGC-[BHQ1]	
*E. canis*	16S rRNA	Ec.139f	CAAATAGTACAAGACGGTAAAGTGCA	[58]
		Ec.32r	AATAGAAGTCTATGTACTTATTTGGA	
		Ec.61p	[FAM]-TAGTGCTGCTTGGGCAACTTTGAGTGAA-[BHQ1]	
TBEV	3′-NCR	F-TBE 1	GGGCGGTTCTTGTTCTCC	[21]
		R-TBE 1	ACACATCACCTCCTTGTCAGACT	
		TBE-Probe-WT	TGAGCCACCATCACCCAGACACA	
*A. phagocytophilum*	16S rRNA	QAP16sf1	TGCCACGGTGAATACGTTCTC	[22]
		QAP16sr1	GCGCACCAGCTTCGAGTT	
		QAP16sr probe	[FAM]-TACACACTGCCCGTCACGCCATG-[BHQ1]	
*Babesia* spp. identification	18S	BabNu2-F	GACACAGGGAGGTAGTGACAAG	[23]
		BabNu2-R	CTAAGAATTTCACCTCTGACAGT	

### Molecular screening of selected pathogens

We used 2× Maxima Probe qPCR Master Mix (Thermo Fischer Scientific) according to the manufacturer’s instructions for DNA detection of *B. burgdorferi* s.l., *B. miyamotoi*, *Anaplasma* spp., *E. canis*, *Ca.* N. mikurensis, and *Babesia* spp. Details on the primers and probes used are described in Table 1. Detection of TBEV RNA was performed with one-step reverse transcriptase PCR (RT-PCR) using TaqMan Fast Virus 1-Step Master Mix (Thermo Fischer Scientific) as described previously [21]. All the reactions included positive and negative controls.

### Identification of *Anaplasma* and *Babesia* species

A differential qPCR [22] and 2× Maxima Probe qPCR Master Mix were used for identification of *A. phagocytophilum*. For identification of *Babesia* species, a traditional PCR protocol [23] using Q5 High-Fidelity PCR Master Mix (New England Biolabs, Inc.) was used. The *Babesia* PCR products were purified with a GeneJET PCR Purification Kit (Thermo Fisher) and sent to the Institute for Molecular Medicine Finland for Sanger sequencing. The *Babesia* species were identified using the BLAST program available at the NCBI.

### 16S next-generation sequencing and analysis

For comparison, we analysed a subset of ticks (*n* = 20) in which pathogens were detected with specific molecular methods, also with a broad-spectrum method, i.e., 16S next-generation sequencing (NGS). To create libraries, the V3–V4 hypervariable region of the prokaryotic 16S RNA sequence was amplified from the DNA extracts as in the 16S Metagenomic Sequencing Library Preparation guide. Sequencing was performed with an Illumina MiSeq instrument using a MiSeq reagent kit v3. The high-quality reads from each sample were analysed using MG-Rast in R [24] together with in-house scripts for taxonomic profiling of the metagenomic data against the Ribosomal Database Project (RDP) database [25].

### Mapping

Occurrence maps of pathogens and tick species were created in Esri ArcGIS (version 10.3.1) (Esri, Redlands, CA, USA). Country boundary shapefiles were downloaded from the open-source spatial database (GADM, 2022).

## Results

### Ticks and tick species

A total of 342 ticks were collected by veterinary clinics and submitted to the University of Helsinki. Ticks were collected from 30 cats and 259 dogs; host species data were missing for eight animals. Most pets were infested by one tick, but some pets had two to four, and one dog even 15 ticks. Of the 303 ticks from which data were available, 224 (73.9%) were attached, including 118 (38.9% of all) engorged ticks. Seventy-nine (26.1%) were crawling on the animal. Life stage was recorded for 206 ticks and included three nymphs (1.5%), 46 adult males (22.3%), and 157 adult females (76.2%).

The tick species was confirmable by both ITS2 qPCR and ITS2 PCR-based sequencing for 271 ticks. Seventy ticks were identified by ITS2 qPCR only, as we were unable to sequence them despite several attempts. One imported tick from Malaga, Spain, was morphologically identified as not being *Ixodes* spp., which was confirmed by sequencing the *ITS2* and *COX1* genes.

**Fig. 2:**
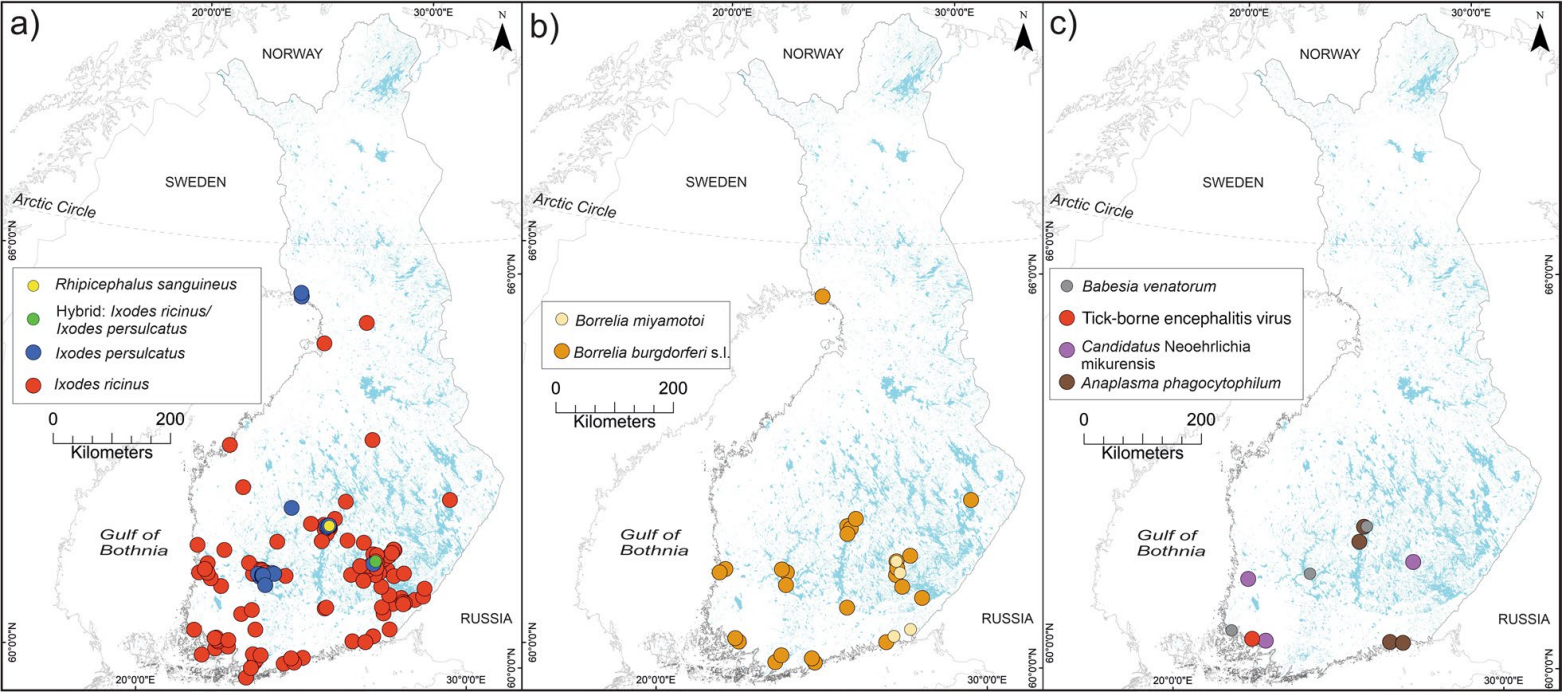
The locations of tick species and detected tick-borne pathogens. Maps indicate the occurrence of confirmed tick species collected from pets (**a**) and pathogens detected in the collected ticks (**b**–**c**)

Figure 2a shows the location and species of ticks collected by the clinics. The molecular identification of tick species revealed that *I. ricinus* (289, 84.5%) was predominant in this collection, followed by *I. persulcatus* (51, 14.9%). One tick (0.3%) showed a hybrid pattern between both species in the duplex qPCR results of the *ITS2* gene, showing two amplification plots for both (*I. ricinus*/*I. persulcatus*), which indicates that the specific probes for the two species were amplified simultaneously. The tick imported from Spain was identified as *Rhipicephalus sanguineus* (0.3%) both morphologically and based on sequencing.

Ticks collected from southern, south-western, western, and eastern Finland were identified as *I. ricinus*. Both *I. ricinus* and *I. persulcatus* were collected from pets in central Finland, which is considered a sympatric area for the two tick species. The northernmost tick samples were collected from Southern Lapland (65.8°N) and were identified as *I. persulcatus*. The ticks were collected over 12 months per clinic, starting from summer of 2020 and ending before the winter of 2021. The earliest ticks in spring were collected on 19 April, and the latest on 24 November.

### Pathogen screening

Details and geographical spread of all pathogens detected in ticks in this study are presented in Table 2 and in Fig. 2b–c. A total of 59 of 342 ticks (17.2%) harboured at least one pathogen.

**Table 2:** Pathogen screening results

Tick species	Pathogens						
	*A. phagocytophilum*	*B. burgdorferi* s.l.	*B. miyamotoi*	*Ca.* N. mikurensis	*E. canis*	*B. venatorum*	Tick-borne encephalitis virus
*I. ricinus*	12	34	5	3	0	3	1
*I. persulcatus*	0	2	0	0	0	0	0
*I. ricinus*/*I. persulcatus*	0	0	0	0	0	0	0
*R. sanguineus*	0	0	0	0	0	1	0
Total (prevalence)	12 (3.51%)	36 (10.5%)	5 (1.46%)	3 (0.88%)	0 (0.0%)	4 (1.17%)	1 (0.3%)
95% confidence interval for prevalence	1.83–6.05	7.48–14.27	0.48–3.38	0.18–2.54	0–1.07	0.32–2.97	0.01–1.62

The most prevalent microbe was *B. burgdorferi* s.l. (36, 10.5%), followed by *Anaplasma* spp. (12, 3.5%), *B. miyamotoi* (5, 1.5%), *Babesia* spp. (4, 1.2%), *Ca*. N. mikurensis (3, 0.9%), and TBEV (1, 0.3%). *Ehrlichia canis* was not detected in this collection. All *Anaplasma* spp. findings were further identified as *A. phagocytophilum*. *Babesia* spp. were confirmed as *Babesia venatorum*. The infection rate differed between the tick species; 56/289 (19.4%) of the *I. ricinus* ticks and 2/51 (3.9%) of *I. persulcatus* were infected. The single *R. sanguineus* was infected with *B. venatorum* (1/1, 100%). The hybrid tick did not show positive results for the tested pathogens (0/1, 0.0%).

Interestingly, the ticks collected from the dog infested by 15 *I. ricinus* ticks carried both *B. burgdorferi* s.l. (four ticks) and *A. phagocytophilum* (three ticks).

### 16S NGS data

To assess the potential of NGS for bacterial detection from tick samples, we used 16S sequencing to detect our specific target pathogens (*Anaplasma*, *Borrelia*, and *Ca*. N. mikurensis) in 20 samples. Only six of these samples produced enough reads for the analysis. The bacterial hits against the sequences in the RDP varied from about 8000 to 130 000 (Additional file 1: Table S1). In these six samples, we detected and confirmed the *Borrelia* and *Ca*. N. mikurensis qPCR findings. In addition, we detected one *Borrelia*-positive sample out of these six samples that was negative in the qPCR. We also confirmed that these six samples with enough reads were *Anaplasma*-negative, confirming the PCR results. Thus, the NGS approach has high potential once optimized for tick samples.

## Discussion

Ticks require blood meals to develop to the next life stage. Consequently, infection rate may vary depending on developmental stage. In *I. ricinus* ticks in Denmark, the infection rate was 2.7 times higher in adults compared to nymphs. Co-infection rates were 12.3% in adult females and 3.5% in nymphs [26]. In our study, 98.5% of the ticks from which data were available were adults. Only 1.5% were nymphs, as these are difficult to detect from pets due to the small size of nymphs and the fur coat of pets, where ticks can easily hide. For the subset of ticks analysed, 38.9% of the ticks were engorged, which, together with the infection rate of 17.2% of these ticks, indicates that these pets were at risk of getting infection. This emphasizes the need for anti-tick medication for pets.

The estimates of tick abundance, microbial content, and infection rate of ticks may differ depending on the collection method [27]. For instance, the commonly used cloth dragging method severely underestimates the abundance of ticks [28]. In Spain, Del Cerro et al. found mostly *Borrelia* spp. and *Rickettsia slovaca* in questing ticks only, while some pathogens, including “*Candidatus* Rickettsia rioja”, *Rickettsia raoultii*, and *A. phagocytophilum* were found in both questing ticks and ticks feeding on animals [27]. Additionally, protozoan pathogens were detected in engorged animal-fed ticks except for *Babesia bigemina*, which was found only in questing ticks collected by dragging [27]. Likewise, in Germany, *Babesia* spp. and *A. phagocytophilum* were most prevalent in engorged ticks collected from roe deer, followed by nymphs and adult questing ticks [29].

Our findings agree with previous studies in Finland, which reported *I. ricinus* and *I. persulcatus* as the prevalent tick species with medical/veterinary importance and their geographical distribution [7, 8, 30]. These two tick species hybridize naturally, as shown by molecular genetic studies [31]. Here, we found one hybrid tick that did not show a positive result for the studied pathogens. We also found one *R. sanguineus* that was positive for *B. venatorum*. It is noteworthy that the dog from which the tick was collected had a travel history with his owner in Spain, explaining its presence in Finland. The risk of importing exotic tick species and pathogens can be increased via traveling with animals without ectoparasitic treatment [10, 32].

Climate and environment play significant roles in the distribution of ticks and tick-borne diseases, as arthropods are especially sensitive to changes in climatic and environmental conditions. Based on the latest climate projections for Finland, mean air temperature is predicted to increase by 2.4 °C in summer and by 3.3 °C in winter by 2070 [33]. Similarly, precipitation is estimated to increase by 5% during summer and by 12% during winter [33]. Warmer temperature and higher precipitation during summer and winter in Finland are expected to impact ticks in several ways. Higher tick abundance, longer activity seasons, and range expansions of both native and invasive tick species are expected to occur. For example, the invasive tick species *Hyalomma marginatum* in migratory birds has already been occasionally reported in Finland [9]. In the other northern European countries, the vector of *Babesia canis*, *Dermacentor reticulatus*, has been observed in dogs and migratory birds [34].

We encountered difficulties in tick species identification. The duplex PCR that we used [17] was suitable to identify *I. ricinus* and *I. persulcatus* but not *R. sanguineus*. Therefore, we performed ITS2 PCR-based sequencing for all ticks. However, we were able to sequence only 79.5% (272) of the ticks. The reason for failure with the remaining 70 ticks may be suboptimal quality of the extracted DNA. For all sequenced DNAs, no incongruence was found between qPCR and ITS2 PCR-based sequencing. For *R. sanguineus*, both methods (ITS2 and COX1 PCR-based sequencing) confirmed its identification. Misidentification of tick species is common. The misidentification rate of ticks collected in different countries and assessed by qualified experts has reached 29.6% [35].

Although information on the prevalence of tick-borne infections in companion animals is limited, infections caused by *Borrelia*, *Anaplasma*, *Babesia,* and TBEV have been reported in Northern European countries [5]. In humans, LB and TBE are the most commonly registered tick-borne diseases in the Nordic countries, including Finland. According to national health care registers, the incidence of microbiologically and clinically confirmed human LB cases is increasing [36]. Our results showed a prevalence of 10.5% for *B. burgdorferi* s.l. in ticks collected from pets in 2020–2021, which is lower than the average prevalence in questing adult ticks of 48.9 ± 8.4% [37]. In Finnish dogs, the seroprevalence of *B. burgdorferi* is low (2.9%) [12], and no antibodies to *B. burgdorferi* were detected in cats. Likewise, another seroprevalence study conducted elsewhere in Europe indicated the rarity of *B. burgdorferi* antibodies in feline samples [38]. Dogs can become infected with *B. burgdorferi* and develop antibodies, but unlike humans, they rarely get sick. The signs in dogs include fever, fatigue, loss of appetite, intermittent lameness, and swollen and painful joints; skin rash is not observed in animals [39].

In contrast to *B. burgdorferi* s.l., *B. miyamotoi* showed a prevalence (1.5%, 95% confidence interval [CI] 0.5–3.4) that is similar to that reported in questing ticks from nationwide studies (0.7%) and in our recent larger collection of ticks from the capital region of Finland (0.6%) [37, 40]. Our previous results also confirmed the circulation of *B. miyamotoi* in ticks from Finland without the detection of bacterial DNA in a large collection of human samples [40]. However, clinical human infections caused by *B. miyamotoi* have been reported elsewhere, confirming its association with human, but not pet animal, disease [41].

We found TBEV in only one tick, representing a low prevalence and corresponding with another local study [37]. The most recent nationwide study on a very large collection of ticks did not detect TBEV in ticks from Finland [37], although the previous nationwide study based on crowdsourcing conducted in 2015 showed a prevalence of 0.2% and 3.0% in *I. ricinus* and *I. persulcatus*, respectively [37]. Overall, TBEV has a very focal distribution in ticks, and extrapolating over larger areas is uncertain. In dogs, TBEV can cause severe and even fatal neurological symptoms, but the high seroprevalence in healthy dogs in some areas, such as in the Åland Islands, indicates that TBEV results mostly in subclinical infection [13]. Further, dogs can be used as sentinels for TBEV and provide an idea for public health surveillance [42].

We detected *A. phagocytophilum* DNA in 12 ticks (3.5%, 95% CI 1.8–6.1), of which three infested a single animal. This prevalence is slightly higher than the prevalence reported in questing ticks (0.6%) [7]. The seroprevalence of *A. phagocytophilum* in Finnish dogs is as high as 5.3%, indicating that infections are common in dogs [12].

*Ehrlichia canis* can cause a serious disease in dogs and is mainly transmitted by *R. sanguineus*, which is not endemic in Finland. In this study, the single *R. sanguineus* tick was imported from Spain, and *E. canis* was not found. We did not detect *E. canis* in other ticks either. Its absence corresponds with the low seroprevalence of *E. canis* (0.3%) in dogs from Finland [12]. Further, the presence of *E. canis* in the most common tick species from the Nordic countries (*Ixodes* sp.) has not been confirmed. However, a low prevalence was documented in *I. ricinus* ticks from the Netherlands [43].

*Babesia* spp. have medical and veterinary importance. According to the Finnish Food Authority, bovine babesiosis was last reported in 2021 [44]. In Finnish humans, a fatal case due to *B. divergens* was reported in a previously ill man who was infected simultaneously with *Borrelia* in 2004 [45]. We are unaware of any other cases at the time of this study. In the current study, *B. venatorum* was found in ticks (three in *I. ricinus* and one in *R. sanguineus*) collected from Taivassalo, Jyväskylä, and Tampere, which are on the southern coast and central part of Finland. *Babesia venatorum* was detected in Finnish ticks collected in 2015 [30], although no human or animal cases have been reported in the country. However, animal and human infections due to *B. venatorum* have been reported elsewhere [46, 47].

The vector of *Babesia canis*, *D. reticulatus*, has not been reported in Finland. However, canine blood samples have confirmed the presence of *B. canis* DNA, and reports have confirmed canine babesiosis in imported dogs [48, 49]. Further, other Nordic countries have confirmed the occasional presence of *D. reticulatus* ticks on dogs, migratory birds, and in nature [34].

In addition to well-known tick-borne pathogens, we studied the prevalence of *Ca.* N. mikurensis, a bacterium emerging in Europe [50]. This bacterium was recently detected in both *I. ricinus* and *I. persulcatus* in Finland [30]. We found a prevalence of 0.9%, which is similar to that reported in *I. ricinus* (0.8%) [7]. *Candidatus* N. mikurensis causes disease in immunocompromised humans and has also been detected in a splenectomized dog [50, 51].

Optimally, follow-up samples would have been available from the animals to study transmission and survey the clinical relevance of the microbial findings. However, such samples were not available in this study and may be available in future studies.

The availability of next-generation technologies means that high-throughput sequencing of the full 16S gene is becoming a reality for species and strain-level bacterial detection and could be used by veterinary laboratories for faster detection of a range of pathogens. Despite the wide use of 16S NGS in bacterial detection, the method still has limitations. For example, the quality and quantity of the extracted DNA may affect the results, especially with hard-bodied ticks that have a thick exoskeleton. Further, the presence of different contaminants (from the kits of extraction or the PCR) can confound the generated data [52]. In our study, as also implied by our inability to fully identify the tick species of a proportion of samples, suboptimal quality of DNA may have limited our NGS findings.

In the future, full-length 16S ribosomal RNA gene sequencing of clinical samples may support species identification and provide important information on bacterial community differences between tick species and geographical locations.

## Conclusions

The infection rate (17.2%) of ticks in this study and the frequency of infestation with engorged or several ticks highlight the risk of pathogen transmission and the need for specific tests when tick-borne infections are suspected and preparedness to treat potentially more serious clinical diseases if multiple pathogens are transmitted. For proper awareness, tick species, tick abundance, and tick-borne pathogens should be surveyed. Pets can be used as sentinels in this regard. Our results emphasize the usefulness of regular veterinary surveillance and need for continuous monitoring for zoonotic infections in humans and animals. We also recommend screening for both known and novel pathogens when suspecting tick-borne infections in humans and animals to avoid misdiagnosis. To control the entry of novel vector species and pathogens, traveling animals should be treated against ectoparasites.

### Supplementary Information

**Additional file 1: Table S1.** Comparison between qPCR and 16S next-generation sequencing results.

## Data Availability

Sequences of *Babesia* species (OR167996–OR167999) and tick species identification by *ITS2* (*I. ricinus* OR204991–OR205210; *I. persulcatus* OR168651–OR168697; *R. sanguineus* OR168702) and *COX1* (*R. sanguineus* OR167605) genes are deposited in GenBank. Other datasets generated during the current study are not publicly available due to the need to protect the privacy of the animal owners, but they are partially available from the corresponding author on reasonable request.
